# Severe Hypertension Leading to Hemorrhagic Stroke in Neurofibromatosis Type 1

**DOI:** 10.7759/cureus.14658

**Published:** 2021-04-24

**Authors:** Mohamed Faris, Michelle Baliss, Robert Coni, Vinod Nambudiri

**Affiliations:** 1 Internal Medicine, Grand Strand Regional Medical Center, Myrtle Beach, USA; 2 Internal Medicine, University of Texas Medical Branch, Galveston, USA; 3 Neurology, Grand Strand Regional Medical Center, Myrtle Beach, USA; 4 Dermatology, Brigham and Women's Hospital, Harvard Medical School, Boston, USA

**Keywords:** neurofibromatosis, hypertension, hypertensive emergency, hemorrhagic, stroke

## Abstract

Neurofibromatosis type 1 (NF-1), also known as von Recklinghausen’s disease, is an autosomal dominant multisystem genetic disorder affecting one in 2,600 individuals. It is caused by a mutation of the NF-1 gene located on chromosome 17q11.2. It is characterized by various cutaneous findings, including cafe-au-lait spots and axillary freckling. Hypertension is a commonly reported finding in adult patients with NF-1 but may also develop during childhood. In most cases, hypertension in NF-1 patients is primary in nature; however, secondary hypertension has been more frequently reported in NF-1 patients due to the association of NF-1 with an increased incidence of pheochromocytomas, bilateral renal artery stenosis, and coarctation of the abdominal aorta. This case reports the consequences of uncontrolled hypertension in a 23-year-old female with NF-1, illustrating the importance of screening for hypertension in children diagnosed with NF-1, and emphasizing the higher incidence of both primary and secondary causes of hypertension in the NF-1 patient population. In this case, no secondary causes of hypertension were found; therefore, a diagnosis of primary hypertension was made and the appropriate therapy was initiated to prevent further complications.

## Introduction

Neurofibromatosis type 1 (NF-1), the most common of three distinct types of neurofibromatosis, is a hereditary autosomal dominant genetic disorder occurring due to mutations in the NF-1 gene on chromosome 17. This gene encodes neurofibromin, a tumor-suppressor protein product widely expressed in many tissue types [1-2]. The severity and clinical manifestations of NF-1 vary significantly among affected individuals. Hypertension is frequently diagnosed in children with NF-1, and in most cases, it is primary in nature [1, 3]. However, NF-1 is associated with a higher incidence of secondary causes of hypertension, most commonly due to vascular abnormalities, such as bilateral renal artery stenosis and coarctation of the abdominal aorta, and less commonly due to pheochromocytomas and certain intracranial tumors [2-3].

The following case discusses the presentation, clinical evaluation, and treatment of a patient with NF-1 complicated by uncontrolled hypertension. The case demonstrates the importance of frequent screening for hypertension in children diagnosed with NF-1, given the higher incidence of primary hypertension and the association with vascular lesions that could predispose NF-1 patients to secondary causes of hypertension. It also highlights the importance of the early initiation of antihypertensives and frequent patient evaluation to ensure that the blood pressure is adequately controlled.

## Case presentation

A 23-year-old female with a history of NF-1 and difficult-to-control hypertension since the age of 12 presented with an acute onset of left-sided weakness, left-sided facial droop, slurred speech, and severe headache. On presentation, her initial blood pressure was markedly elevated at 240/140 mmHg with otherwise normal vital signs. The patient admitted to non-compliance with her blood pressure medications during the two weeks before presentation. She denied smoking, alcohol use, and illicit drug use. She is a college student with a known family history of NF-1 and had an optic glioma diagnosed at the age of five. There was no family history of multiple endocrine neoplasias.

**Figure 1:**
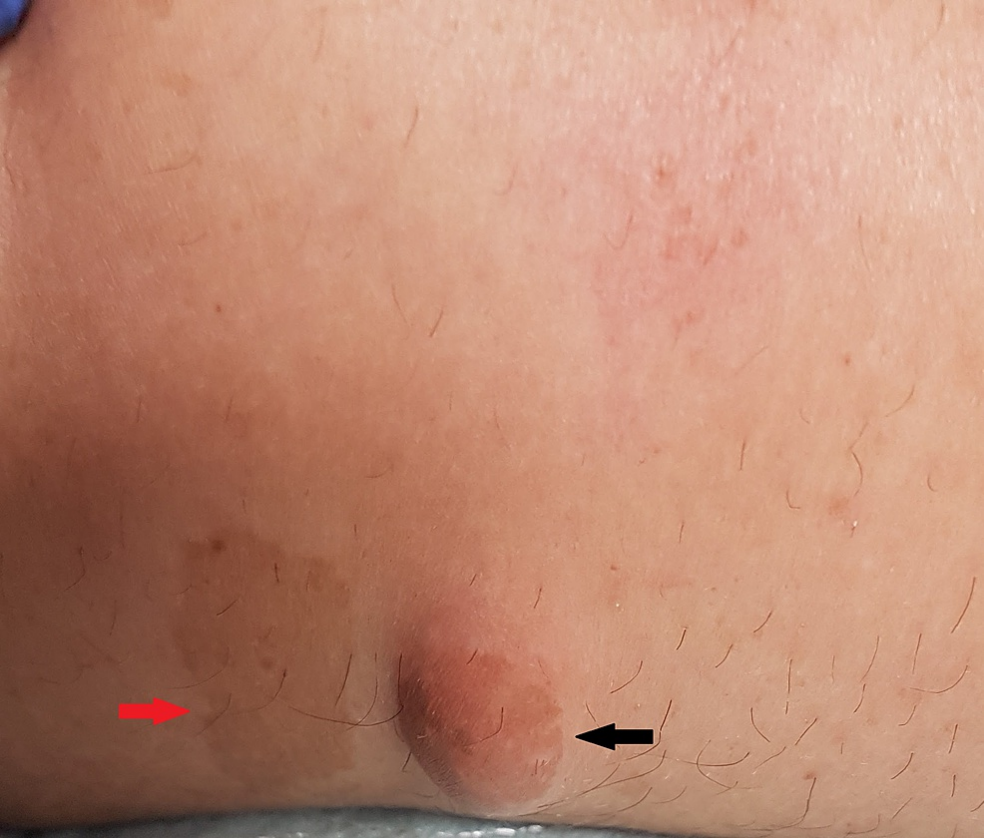
Plexiform neuroma (black arrow), cafe-au-lait macule (red arrow)

**Figure 2:**
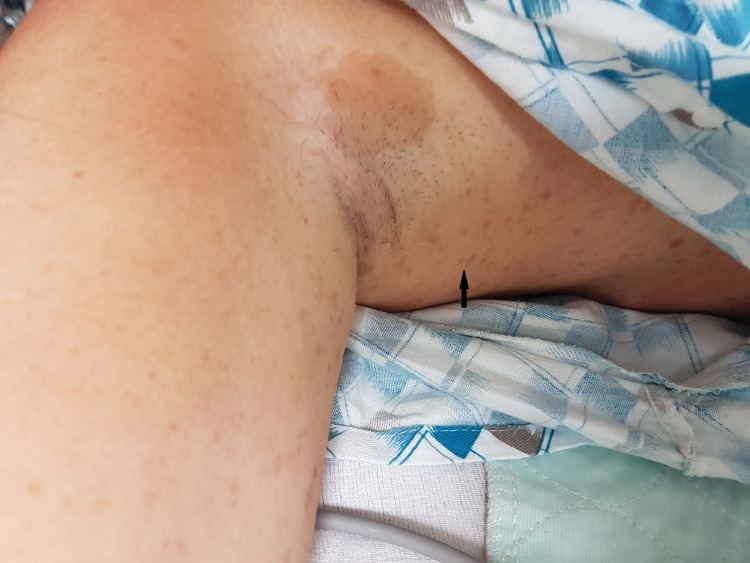
Axillary freckling (black arrow)

Physical examination was notable for skin findings, including multiple widespread large cafe-au-lait macules, neurofibromas, and axillary freckling, as well as multiple pigmented iris lesions consistent with Lisch nodules (Figures 1-2). The patient appeared lethargic, had a blunted affect, and was alert and oriented to person, place, and time. Further neurological examination revealed left-sided nasolabial fold flattening, left-sided paralysis and sensory loss, left-sided hemineglect, and a positive Babinski reflex on the left foot. No abdominal bruits were appreciated on the physical examination.

**Figure 3:**
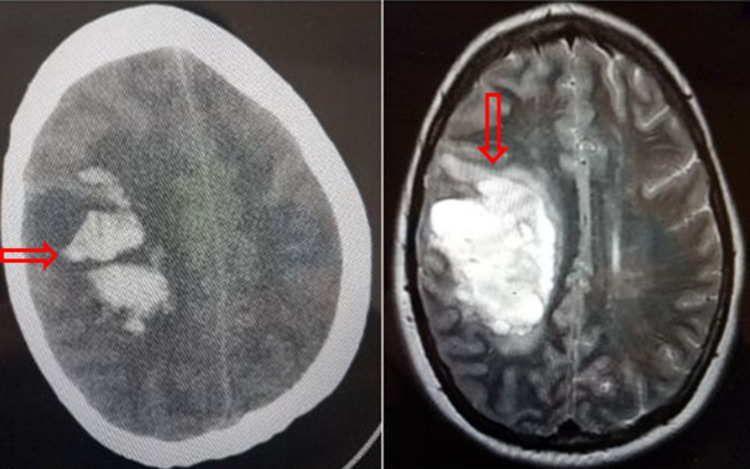
CT scan of the head (left) and MRI of the brain (right), both showing large multifocal intraparenchymal hemorrhage (red arrows) of the right frontal and parietal lobe with associated edema and mass effect causing subfalcine herniation and rightward transtentorial herniation CT: computed tomography; MRI: magnetic resonance imaging

Given her neurologic findings on presentation, a non-contrast computed tomography (CT) scan of the head and a magnetic resonance imaging (MRI) scan of the brain were performed, both revealing a hemorrhagic cerebrovascular accident affecting her right parietal lobe and right basal ganglia (Figure 3).

Blood pressure control was initiated and ultimately required the use of several antihypertensive medications, including labetalol, nicardipine, lisinopril, and clonidine.

To workup secondary causes of her hypertension, urinary catecholamines and metanephrines were measured, and CT and MRI of the head, neck, and abdomen were performed. A magnetic resonance angiography of the brain done one year earlier in another hospital was reviewed; all were negative for possible tumor, vascular structural abnormality, or other contributory etiology. The patient’s blood pressure eventually decreased over the course of her hospital stay to 140/90 mmHg, and she was discharged to a stroke rehabilitation facility.

## Discussion

NF-1 is an autosomal dominant disorder with a prevalence of one in 3,000 caused by germline inactivating mutation in the NF1 gene on chromosome 17 that is characterized by various cutaneous and neurological manifestations [2]. Two or more of the following major criteria must be met to make a diagnosis of NF-1 in a patient: six or more café-au-lait macules, axillary or inguinal freckling, two or more cutaneous neurofibromas, one plexiform neurofibroma, characteristic bony lesions (pseudarthrosis, sphenoid wing hypoplasia), an optic glioma, two or more iris Lisch nodules, or a first-degree relative with NF-1 [4].

The patient met the diagnostic criteria of NF-1 at age five when she presented with headaches and was found to have an optic glioma, in addition to having a family history of NF-1 in her mother and two sisters. She later developed multiple café-au-lait macules and patches, neurofibromas, axillary freckling, and multiple Lisch nodules, which further supported her diagnosis.

NF-1 is associated with the development of hypertension in approximately 16% of patients, which may be primary or secondary [3, 5]. Hypertension in NF-1 may develop at any age and, in most cases, is essential in nature [1]. Patients with NF-1 have a higher incidence of bilateral renal artery stenosis, pheochromocytoma, and coarctation of the abdominal aorta than the general population [3-4]. NF-1 is associated with a 0.1% - 5.7% chance of developing a pheochromocytoma, which is significantly higher than the 0.0008% chance found in the general population [4, 6]. Fifteen percent of adults and 30% of childhood pheochromocytomas are extra-adrenal [7]. The association of NF-1 with multiple conditions related to hypertension makes the evaluation of hypertension in patients with NF-1 a noteworthy diagnostic challenge.

In this case, no identifiable cause for secondary hypertension was appreciated on the various laboratory and imaging studies done for this patient. Thus, a diagnosis of essential hypertension was established. Even though essential hypertension is the most common cause of hypertension in patients with NF-1, the above-average incidence of pheochromocytoma and bilateral renal artery stenosis in NF-1 patients warrants intensive investigation for the diagnosis of these disorders as possible causes of secondary hypertension.

## Conclusions

This case illustrates the importance of recognizing and managing hypertension in patients with NF-1, given the potentially devastating sequelae of hemorrhagic strokes at a young age. The association of NF-1 with pheochromocytomas, bilateral renal artery stenosis, and coarctation of the abdominal aorta should prompt evaluation for these possibilities in patients with NF-1 to ensure the institution of appropriate treatments and prophylaxis. Frequent screening for hypertension in children diagnosed with NF-1 is important, given the higher incidence of primary hypertension.
